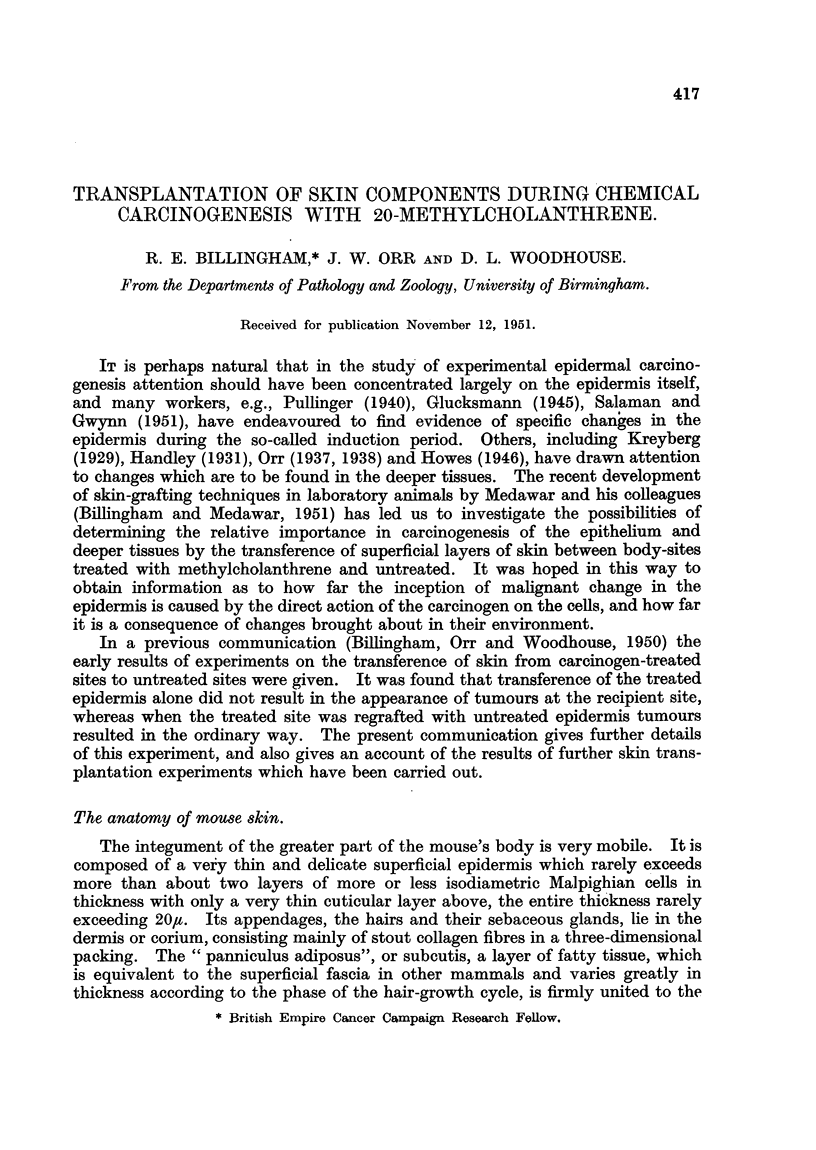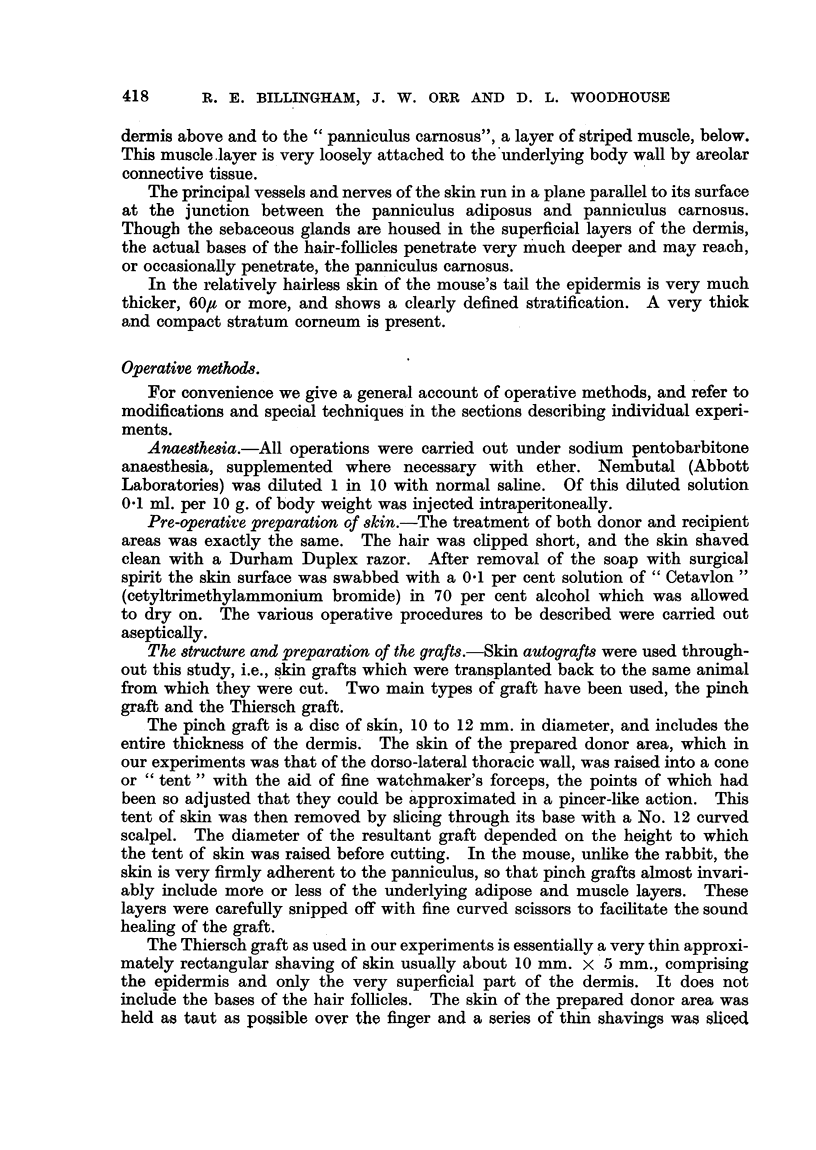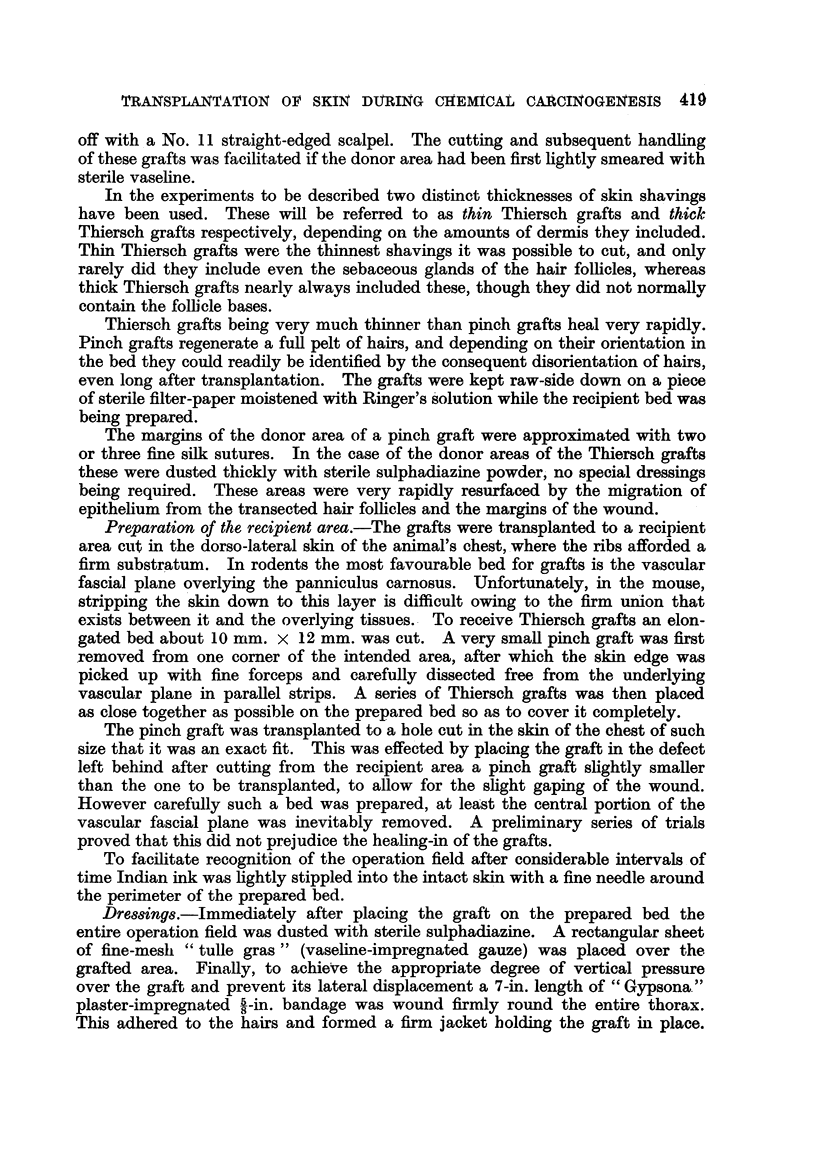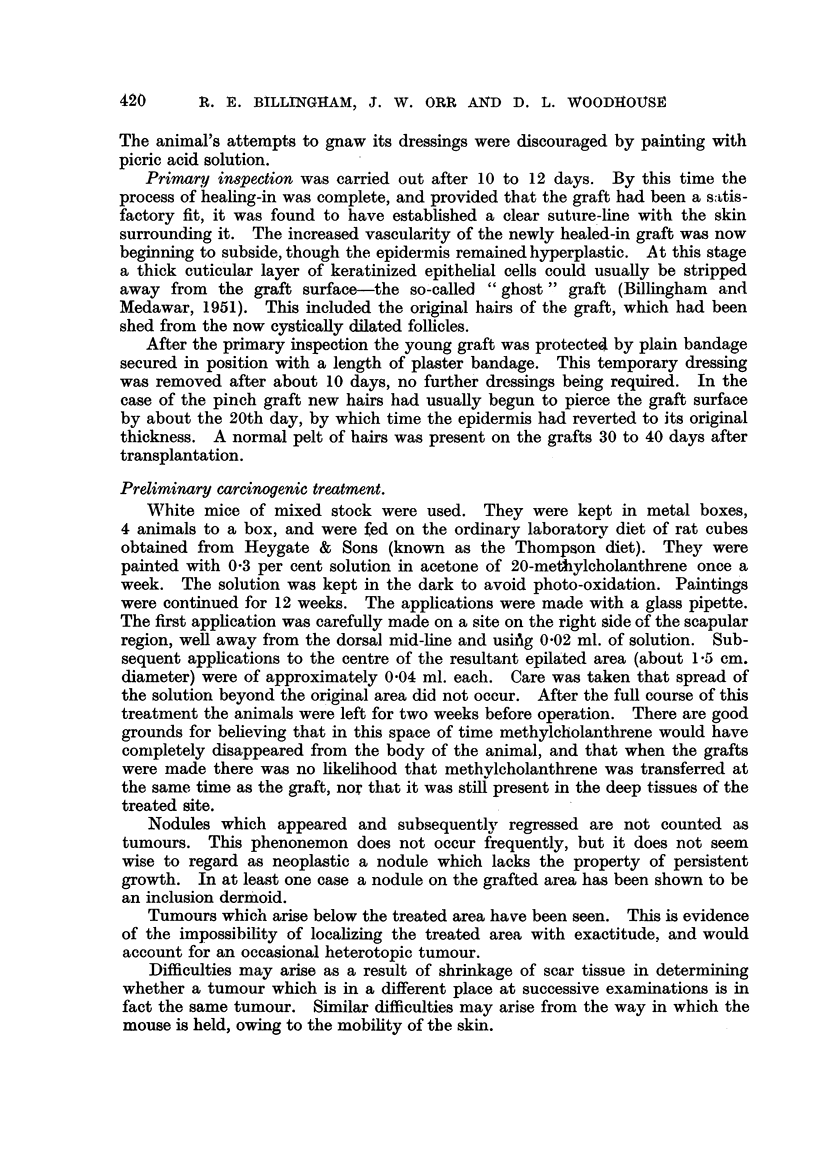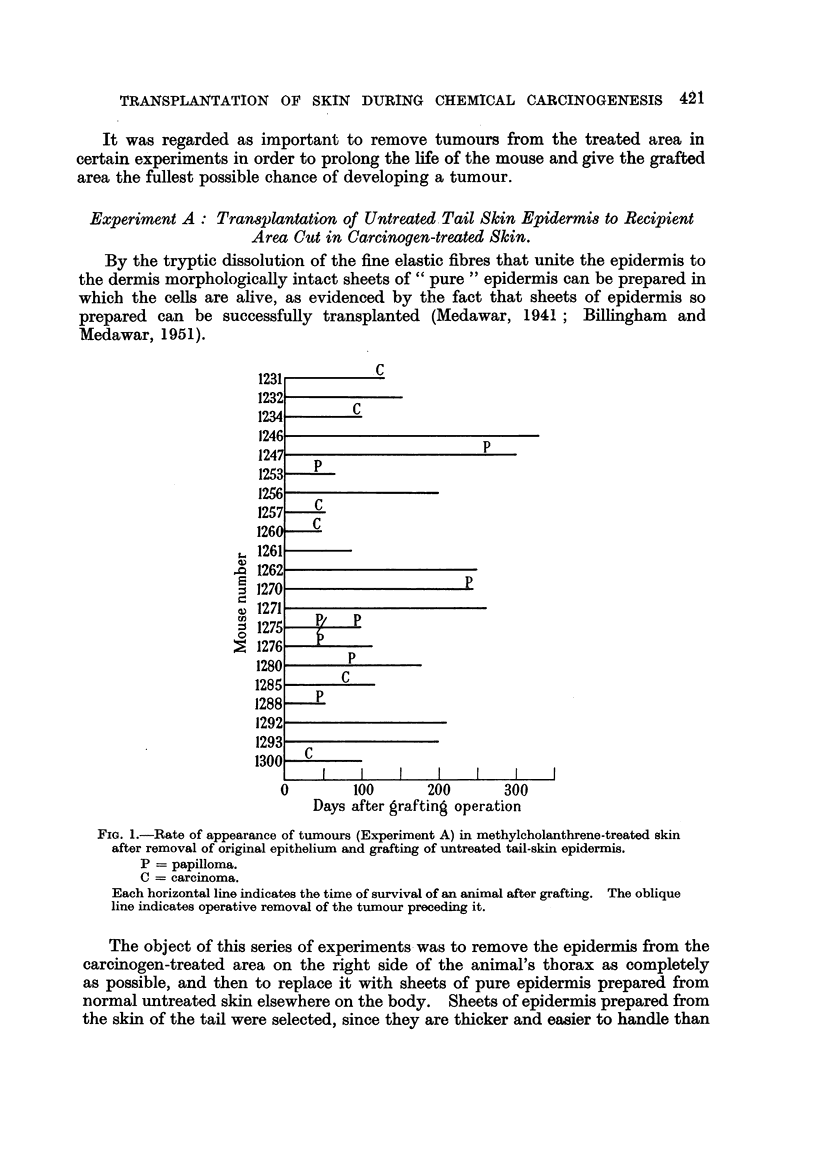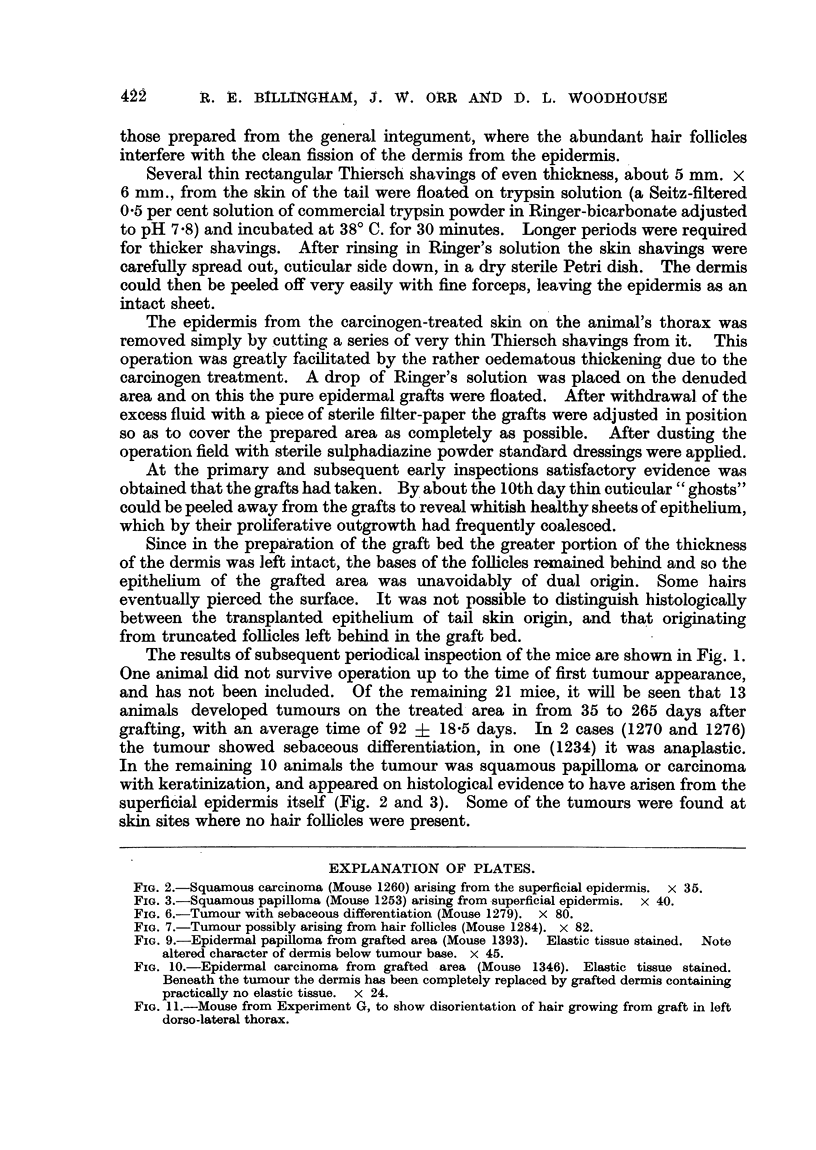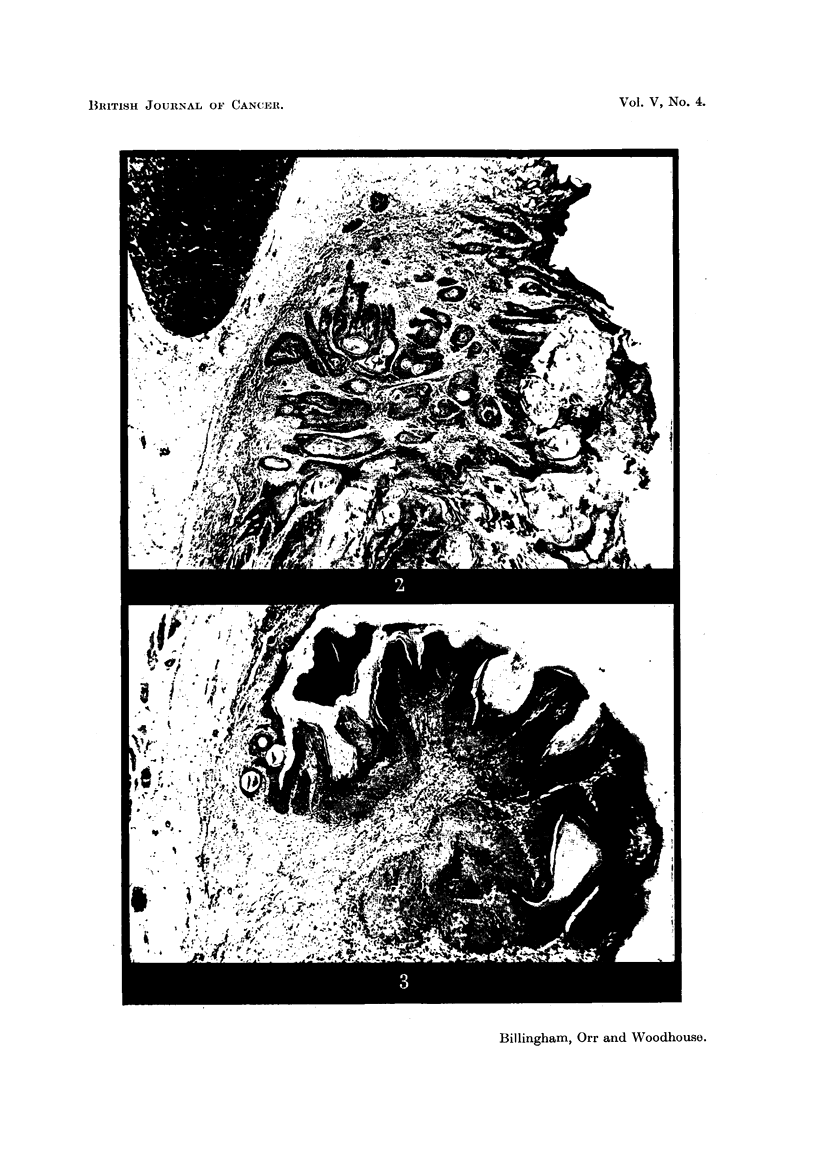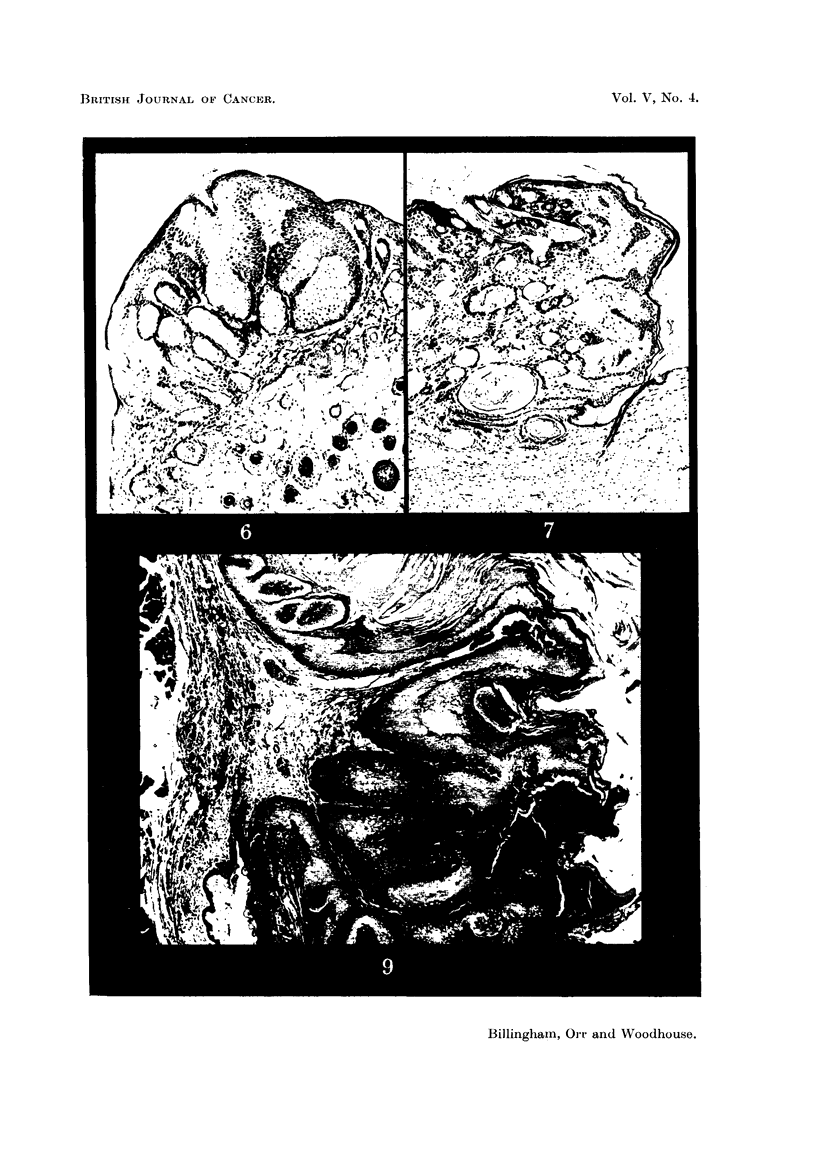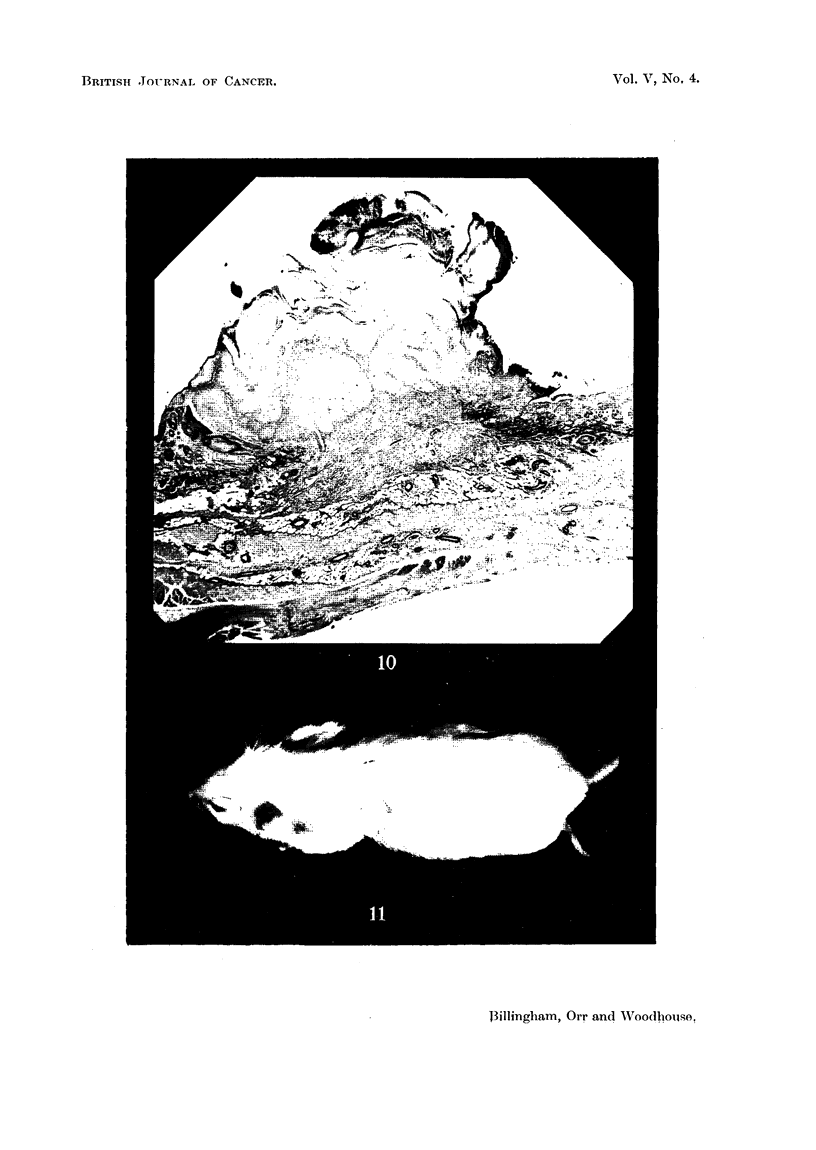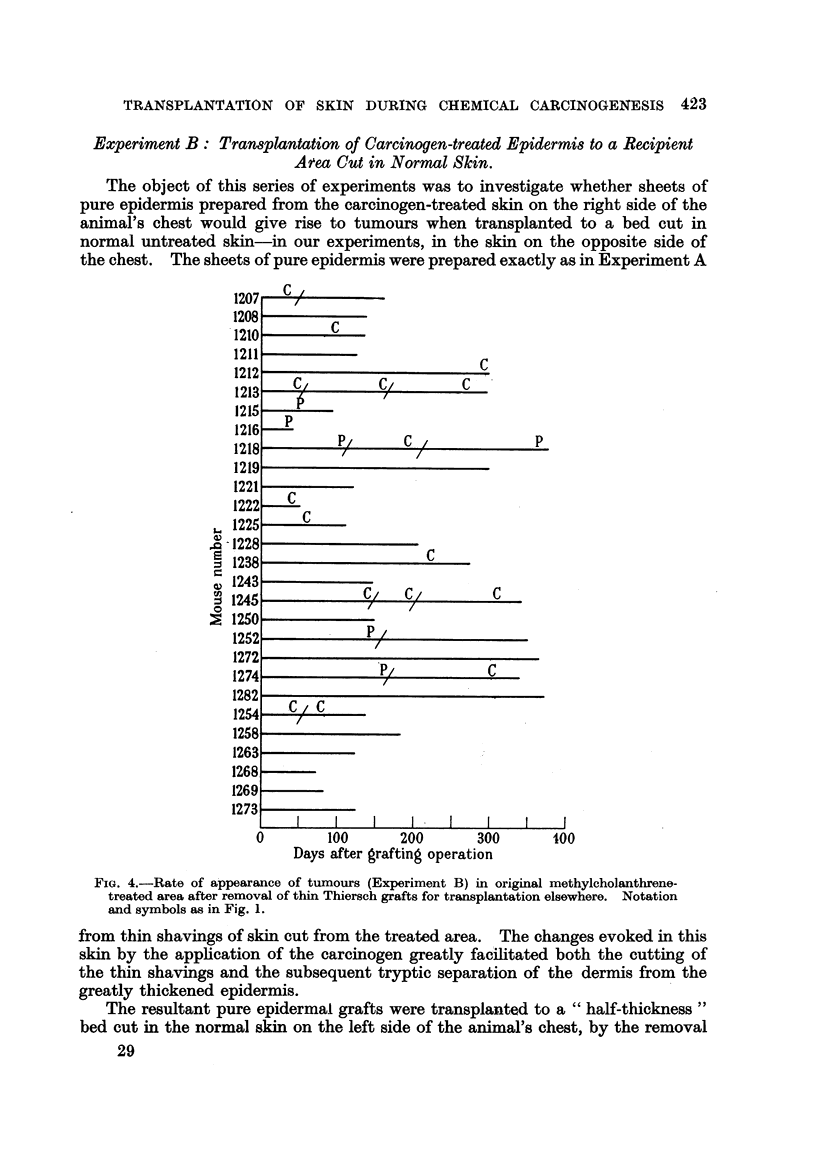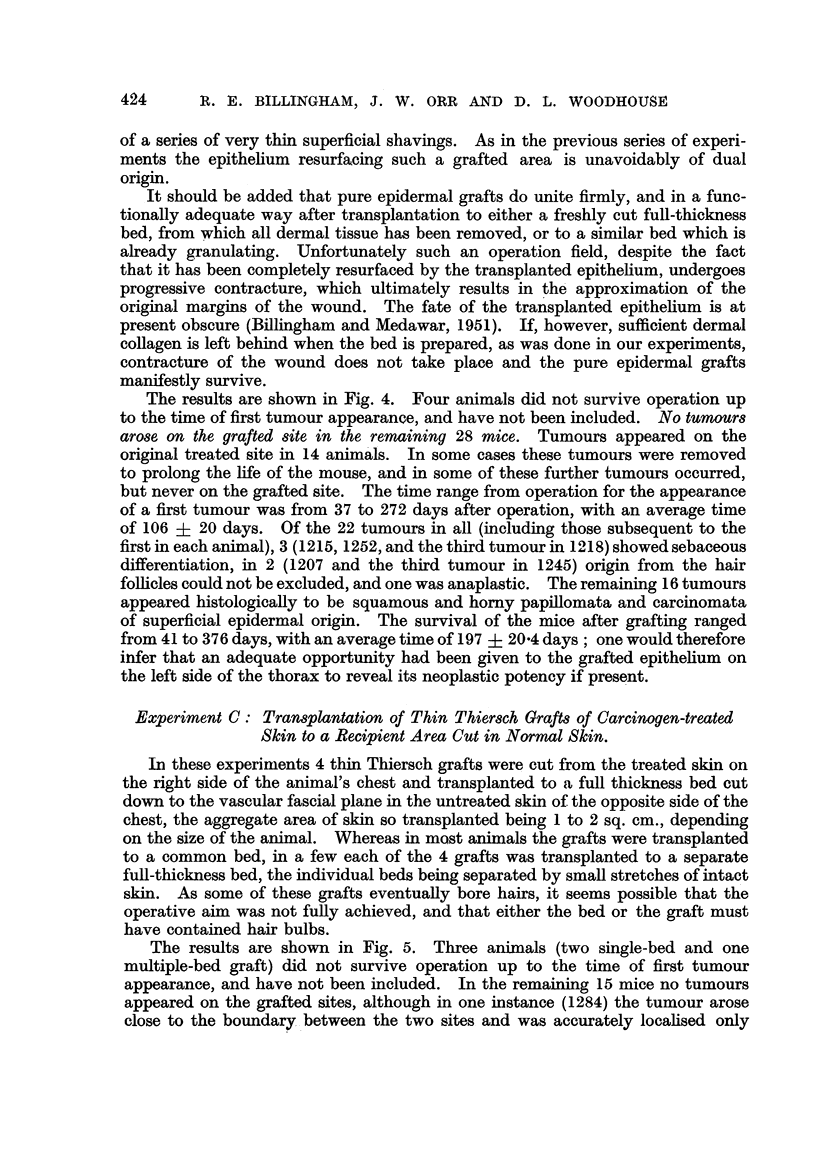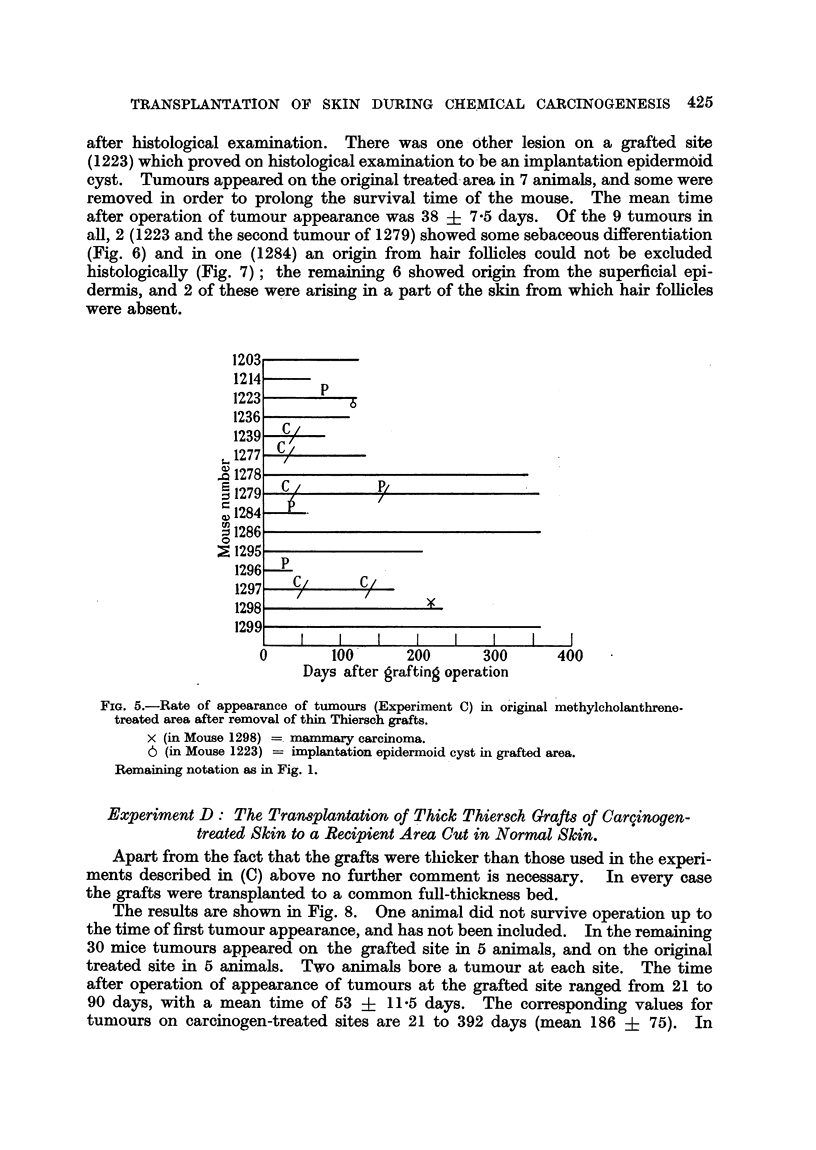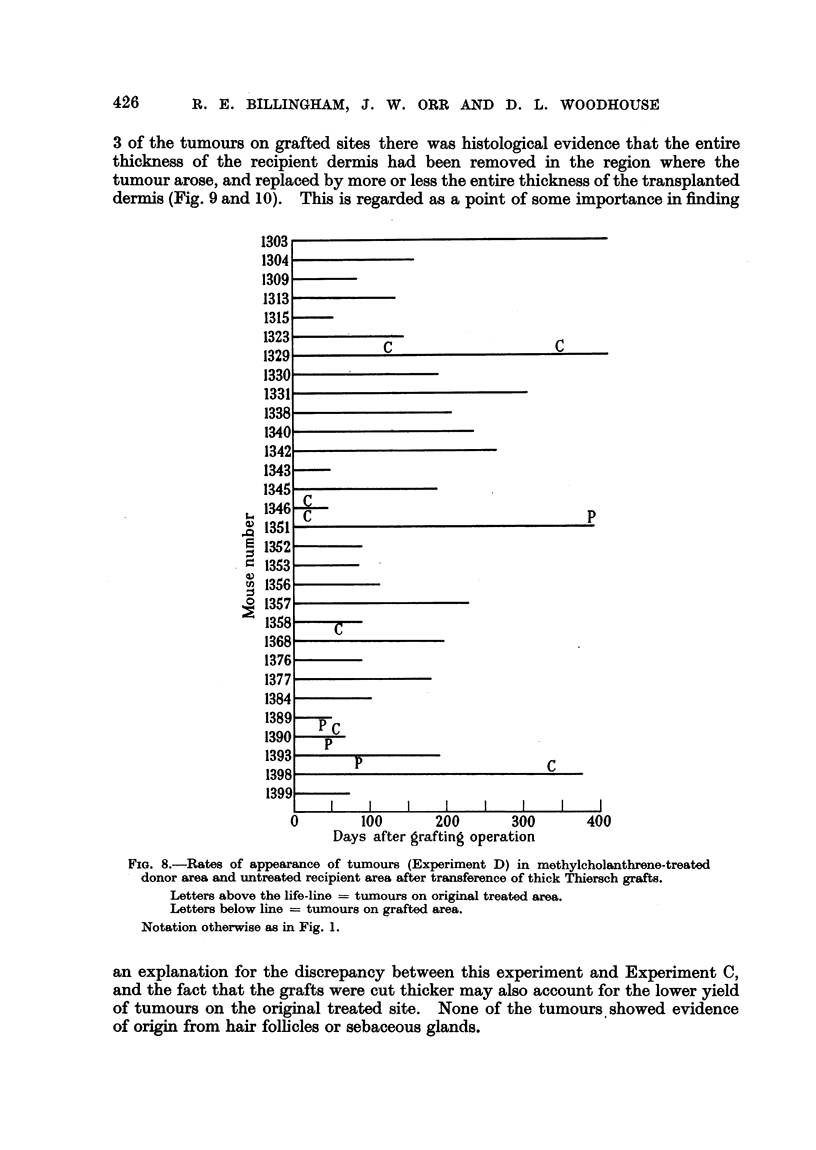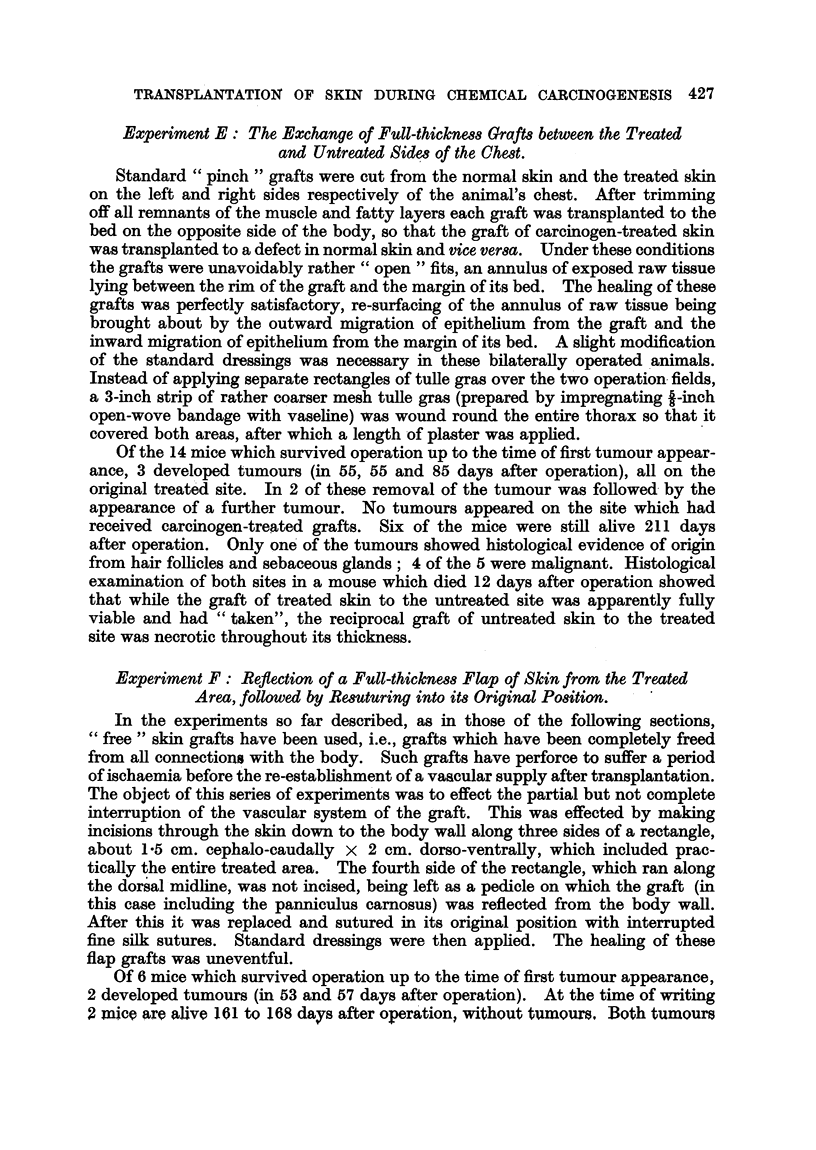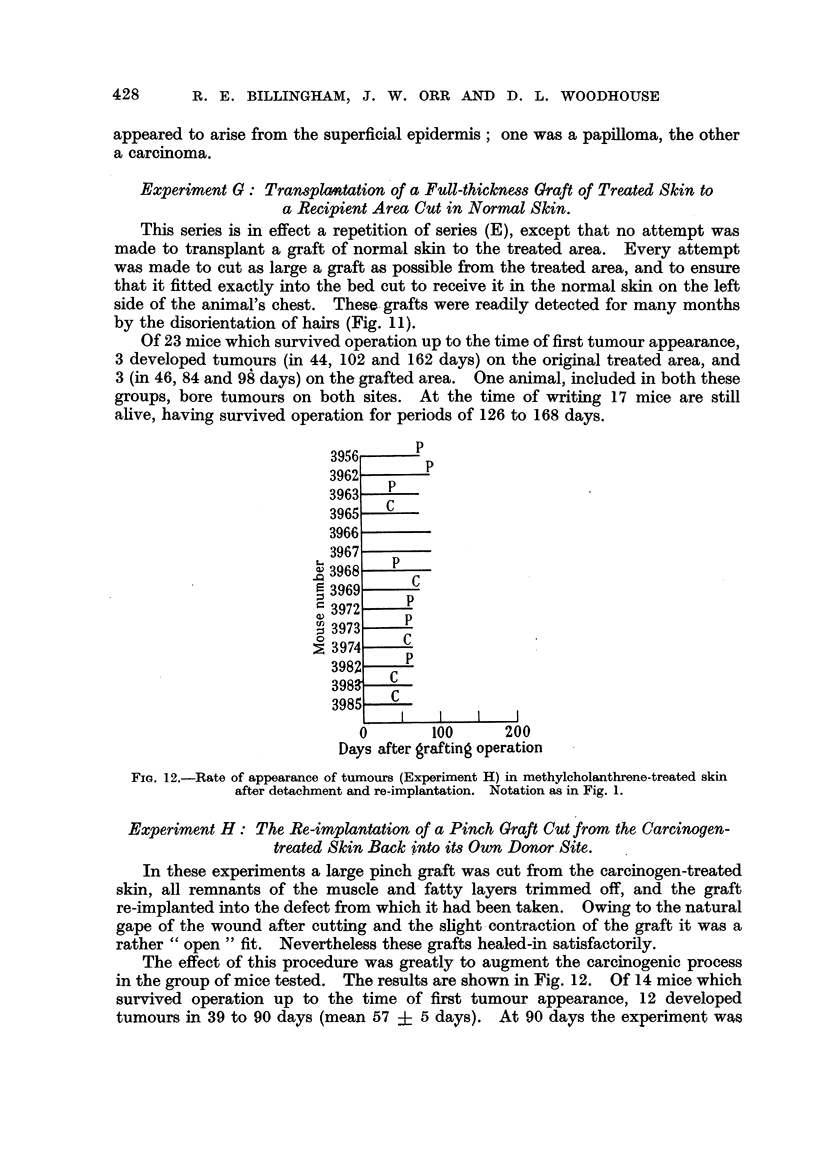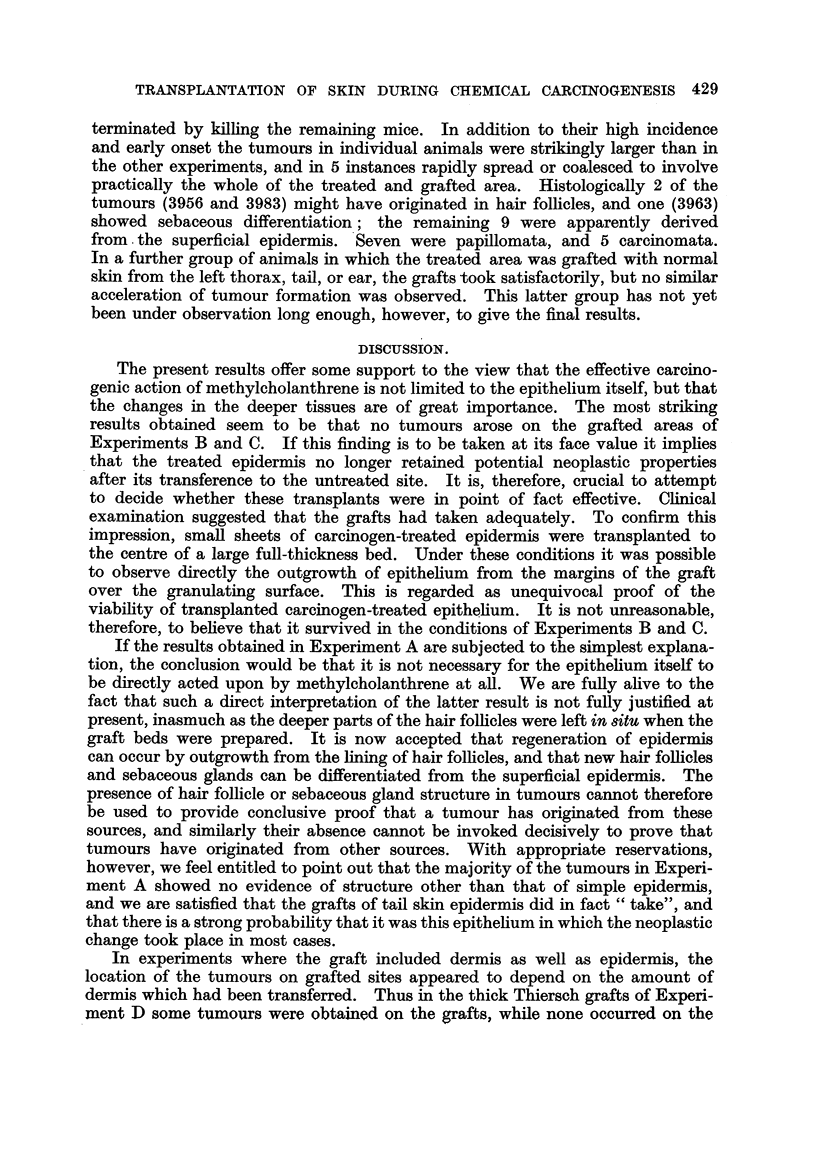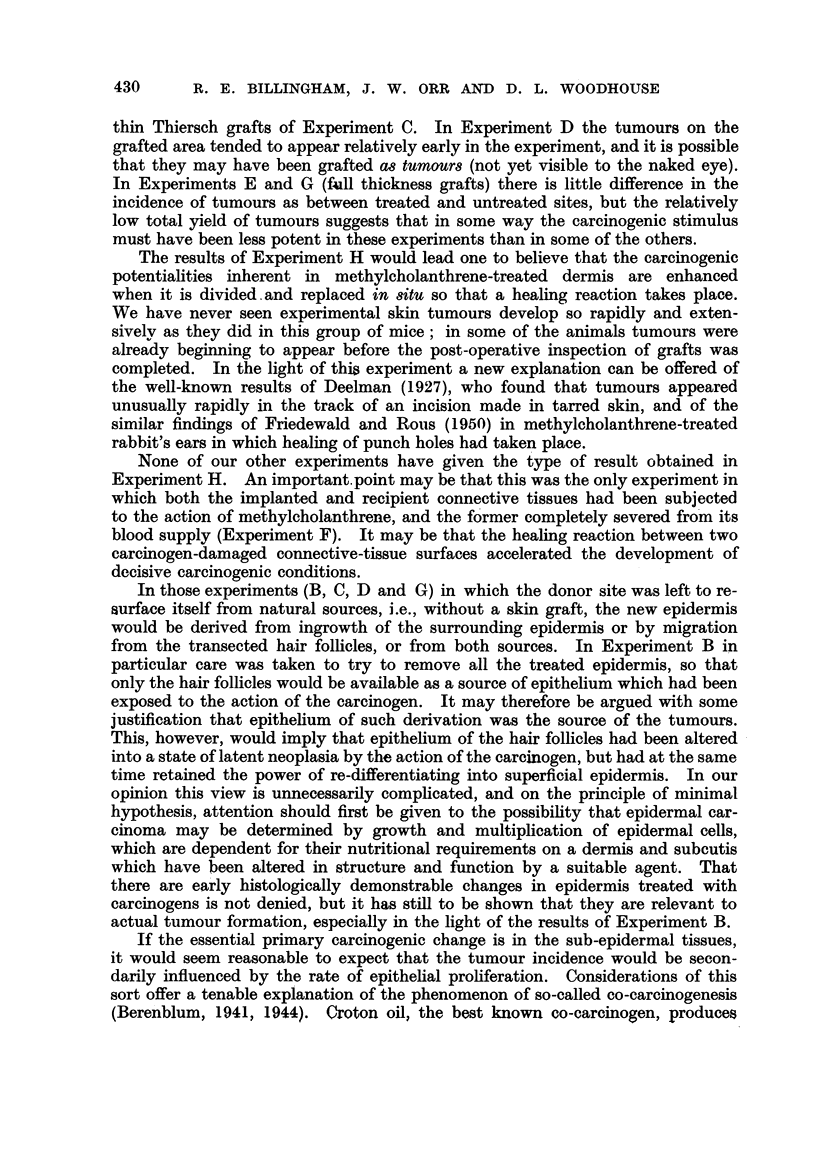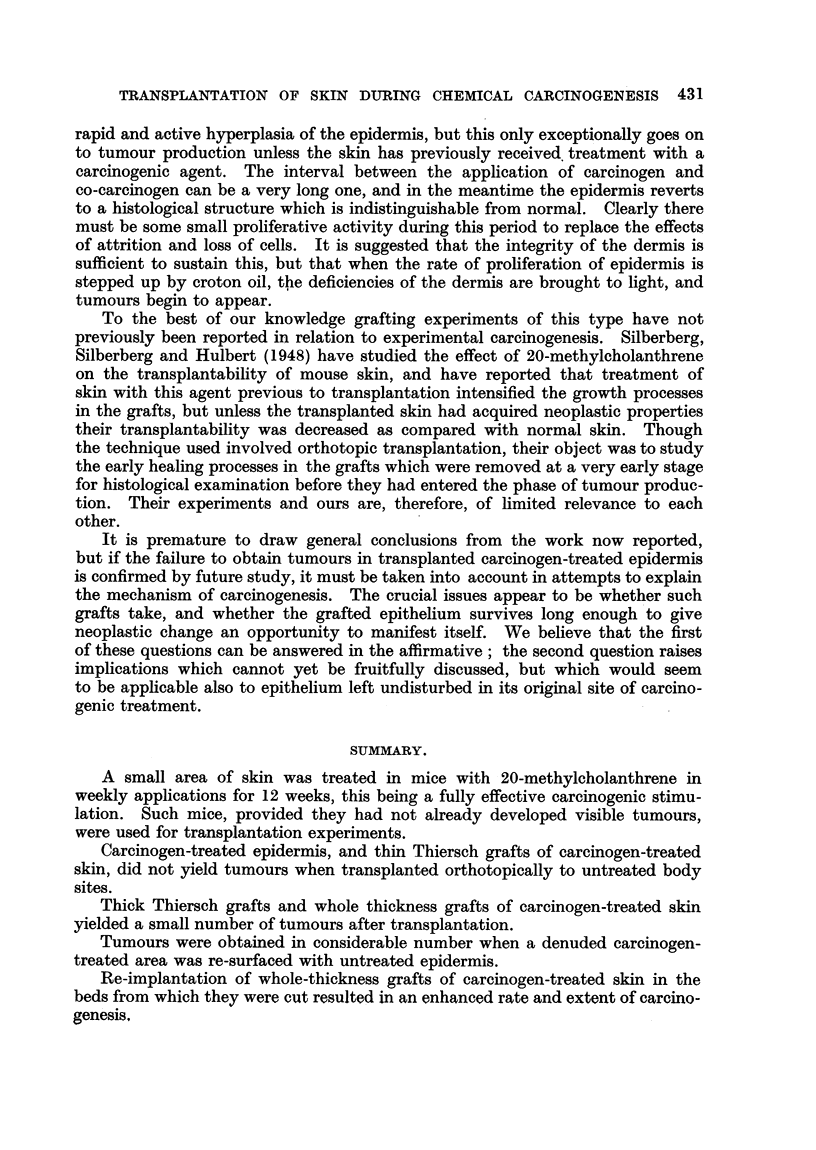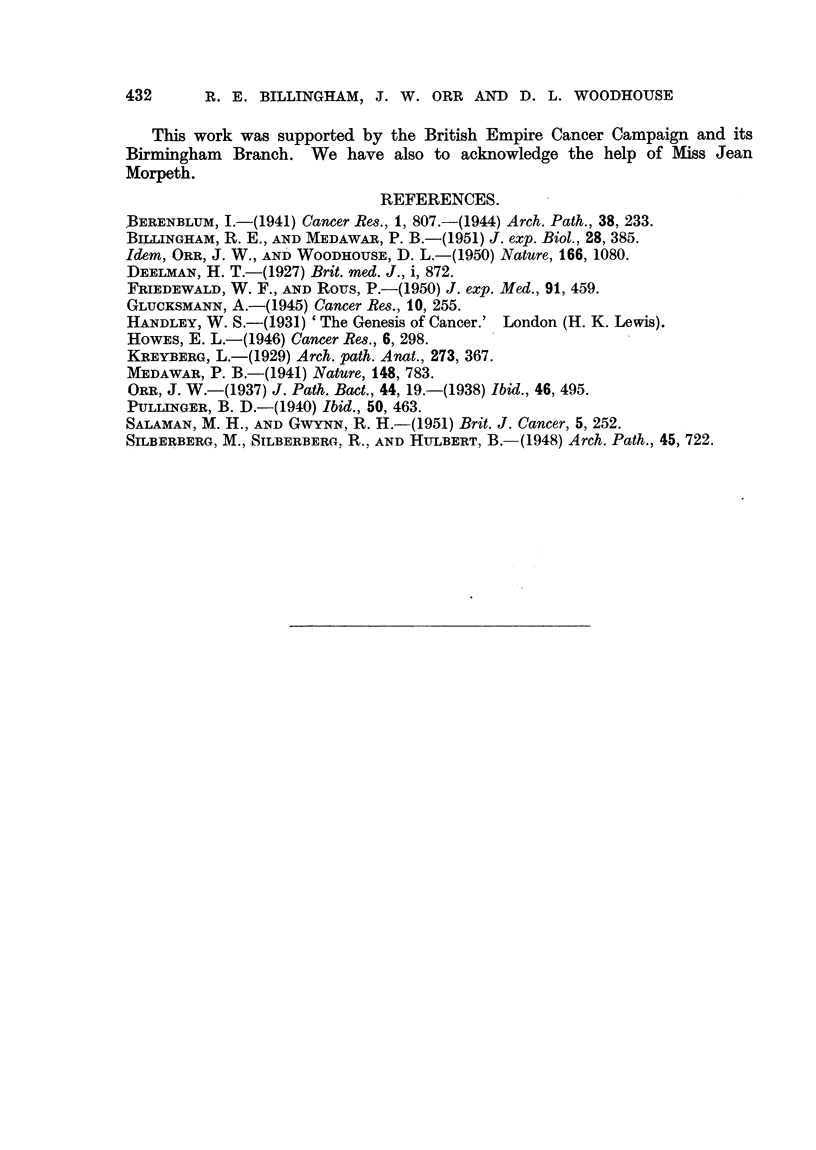# Transplantation of Skin Components during Chemical Carcinogenesis with 20-Methylcholanthrene

**DOI:** 10.1038/bjc.1951.48

**Published:** 1951-12

**Authors:** R. E. Billingham, J. W. Orr, D. L. Woodhouse

## Abstract

**Images:**


### TRANSPLANTATION OF SKIN COMPONENTS DURING CHEMICAL CARCINOGENESIS WITH 20-METHYLCHOLANTHRENE.

R. E. BILLINGHAM,* J. W. ORR AND D. L. WOODHOUSE.

*From the Departments of Pathology and Zoology, University of Birmingham.*

Received for publication November 12, 1951.

IT is perhaps natural that in the study of experimental epidermal carcinogenesis attention should have been concentrated largely on the epidermis itself, and many workers, e.g., Pullinger (1940), Glucksmann (1945), Salaman and Gwynn (1951), have endeavoured to find evidence of specific changes in the epidermis during the so-called induction period. Others, including Kreyberg (1929), Handley (1931), Orr (1937, 1938) and Howes (1946), have drawn attention to changes which are to be found in the deeper tissues. The recent development of skin-grafting techniques in laboratory animals by Medawar and his colleagues (Billingham and Medawar, 1951) has led us to investigate the possibilities of determining the relative importance in carcinogenesis of the epithelium and deeper tissues by the transference of superficial layers of skin between body-sites treated with methylcholanthrene and untreated. It was hoped in this way to obtain information as to how far the inception of malignant change in the epidermis is caused by the direct action of the carcinogen on the cells, and how far it is a consequence of changes brought about in their environment.

In a previous communication (Billingham, Orr and Woodhouse, 1950) the early results of experiments on the transference of skin from carcinogen-treated sites to untreated sites were given. It was found that transference of the treated epidermis alone did not result in the appearance of tumours at the recipient site, whereas when the treated site was regrafted with untreated epidermis tumours resulted in the ordinary way. The present communication gives further details of this experiment, and also gives an account of the results of further skin transplantation experiments which have been carried out.

### *The anatomy of mouse skin.*

The integument of the greater part of the mouse's body is very mobile. It is composed of a very thin and delicate superficial epidermis which rarely exceeds more than about two layers of more or less isodiametric Malpighian cells in thickness with only a very thin cuticular layer above, the entire thickness rarely exceeding $20\mu$. Its appendages, the hairs and their sebaceous glands, lie in the dermis or corium, consisting mainly of stout collagen fibres in a three-dimensional packing. The " panniculus adiposus", or subcutis, a layer of fatty tissue, which is equivalent to the superficial fascia in other mammals and varies greatly in thickness according to the phase of the hair-growth cycle, is firmly united to the

* British Empire Cancer Campaign Research Fellow.

dermis above and to the " panniculus carnosus", a layer of striped muscle, below. This muscle layer is very loosely attached to the underlying body wall by areolar connective tissue.

The principal vessels and nerves of the skin run in a plane parallel to its surface at the junction between the panniculus adiposus and panniculus carnosus. Though the sebaceous glands are housed in the superficial layers of the dermis, the actual bases of the hair-follicles penetrate very much deeper and may reach, or occasionally penetrate, the panniculus carnosus.

In the relatively hairless skin of the mouse's tail the epidermis is very much thicker, $60\mu$ or more, and shows a clearly defined stratification. A very thick and compact stratum corneum is present.

*Operative methods.*

For convenience we give a general account of operative methods, and refer to modifications and special techniques in the sections describing individual experiments.

*Anaesthesia.*—All operations were carried out under sodium pentobarbitone anaesthesia, supplemented where necessary with ether. Nembutal (Abbott Laboratories) was diluted 1 in 10 with normal saline. Of this diluted solution 0·1 ml. per 10 g. of body weight was injected intraperitoneally.

*Pre-operative preparation of skin.*—The treatment of both donor and recipient areas was exactly the same. The hair was clipped short, and the skin shaved clean with a Durham Duplex razor. After removal of the soap with surgical spirit the skin surface was swabbed with a 0·1 per cent solution of " Cetavlon " (cetyltrimethylammonium bromide) in 70 per cent alcohol which was allowed to dry on. The various operative procedures to be described were carried out aseptically.

*The structure and preparation of the grafts.*—Skin *autografts* were used throughout this study, i.e., skin grafts which were transplanted back to the same animal from which they were cut. Two main types of graft have been used, the pinch graft and the Thiersch graft.

The pinch graft is a disc of skin, 10 to 12 mm. in diameter, and includes the entire thickness of the dermis. The skin of the prepared donor area, which in our experiments was that of the dorso-lateral thoracic wall, was raised into a cone or " tent " with the aid of fine watchmaker's forceps, the points of which had been so adjusted that they could be approximated in a pincer-like action. This tent of skin was then removed by slicing through its base with a No. 12 curved scalpel. The diameter of the resultant graft depended on the height to which the tent of skin was raised before cutting. In the mouse, unlike the rabbit, the skin is very firmly adherent to the panniculus, so that pinch grafts almost invariably include more or less of the underlying adipose and muscle layers. These layers were carefully snipped off with fine curved scissors to facilitate the sound healing of the graft.

The Thiersch graft as used in our experiments is essentially a very thin approximately rectangular shaving of skin usually about 10 mm. $\times$ 5 mm., comprising the epidermis and only the very superficial part of the dermis. It does not include the bases of the hair follicles. The skin of the prepared donor area was held as taut as possible over the finger and a series of thin shavings was sliced off with a No. 11 straight-edged scalpel. The cutting and subsequent handling of these grafts was facilitated if the donor area had been first lightly smeared with sterile vaseline.

In the experiments to be described two distinct thicknesses of skin shavings have been used. These will be referred to as *thin* Thiersch grafts and *thick* Thiersch grafts respectively, depending on the amounts of dermis they included. Thin Thiersch grafts were the thinnest shavings it was possible to cut, and only rarely did they include even the sebaceous glands of the hair follicles, whereas thick Thiersch grafts nearly always included these, though they did not normally contain the follicle bases.

Thiersch grafts being very much thinner than pinch grafts heal very rapidly. Pinch grafts regenerate a full pelt of hairs, and depending on their orientation in the bed they could readily be identified by the consequent disorientation of hairs, even long after transplantation. The grafts were kept raw-side down on a piece of sterile filter-paper moistened with Ringer's solution while the recipient bed was being prepared.

The margins of the donor area of a pinch graft were approximated with two or three fine silk sutures. In the case of the donor areas of the Thiersch grafts these were dusted thickly with sterile sulphadiazine powder, no special dressings being required. These areas were very rapidly resurfaced by the migration of epithelium from the transected hair follicles and the margins of the wound.

*Preparation of the recipient area.*—The grafts were transplanted to a recipient area cut in the dorso-lateral skin of the animal's chest, where the ribs afforded a firm substratum. In rodents the most favourable bed for grafts is the vascular fascial plane overlying the panniculus carnosus. Unfortunately, in the mouse, stripping the skin down to this layer is difficult owing to the firm union that exists between it and the overlying tissues. To receive Thiersch grafts an elongated bed about 10 mm. × 12 mm. was cut. A very small pinch graft was first removed from one corner of the intended area, after which the skin edge was picked up with fine forceps and carefully dissected free from the underlying vascular plane in parallel strips. A series of Thiersch grafts was then placed as close together as possible on the prepared bed so as to cover it completely.

The pinch graft was transplanted to a hole cut in the skin of the chest of such size that it was an exact fit. This was effected by placing the graft in the defect left behind after cutting from the recipient area a pinch graft slightly smaller than the one to be transplanted, to allow for the slight gaping of the wound. However carefully such a bed was prepared, at least the central portion of the vascular fascial plane was inevitably removed. A preliminary series of trials proved that this did not prejudice the healing-in of the grafts.

To facilitate recognition of the operation field after considerable intervals of time Indian ink was lightly stippled into the intact skin with a fine needle around the perimeter of the prepared bed.

*Dressings.*—Immediately after placing the graft on the prepared bed the entire operation field was dusted with sterile sulphadiazine. A rectangular sheet of fine-mesh "tulle gras" (vaseline-impregnated gauze) was placed over the grafted area. Finally, to achieve the appropriate degree of vertical pressure over the graft and prevent its lateral displacement a 7-in. length of "Gypsona" plaster-impregnated $\frac{5}{8}$-in. bandage was wound firmly round the entire thorax. This adhered to the hairs and formed a firm jacket holding the graft in place.

The animal's attempts to gnaw its dressings were discouraged by painting with picric acid solution.

*Primary inspection* was carried out after 10 to 12 days. By this time the process of healing-in was complete, and provided that the graft had been a satisfactory fit, it was found to have established a clear suture-line with the skin surrounding it. The increased vascularity of the newly healed-in graft was now beginning to subside, though the epidermis remained hyperplastic. At this stage a thick cuticular layer of keratinized epithelial cells could usually be stripped away from the graft surface—the so-called "ghost" graft (Billingham and Medawar, 1951). This included the original hairs of the graft, which had been shed from the now cystically dilated follicles.

After the primary inspection the young graft was protected by plain bandage secured in position with a length of plaster bandage. This temporary dressing was removed after about 10 days, no further dressings being required. In the case of the pinch graft new hairs had usually begun to pierce the graft surface by about the 20th day, by which time the epidermis had reverted to its original thickness. A normal pelt of hairs was present on the grafts 30 to 40 days after transplantation.

*Preliminary carcinogenic treatment.*

White mice of mixed stock were used. They were kept in metal boxes, 4 animals to a box, and were fed on the ordinary laboratory diet of rat cubes obtained from Heygate & Sons (known as the Thompson diet). They were painted with 0·3 per cent solution in acetone of 20-methylcholanthrene once a week. The solution was kept in the dark to avoid photo-oxidation. Paintings were continued for 12 weeks. The applications were made with a glass pipette. The first application was carefully made on a site on the right side of the scapular region, well away from the dorsal mid-line and using 0·02 ml. of solution. Subsequent applications to the centre of the resultant epilated area (about 1·5 cm. diameter) were of approximately 0·04 ml. each. Care was taken that spread of the solution beyond the original area did not occur. After the full course of this treatment the animals were left for two weeks before operation. There are good grounds for believing that in this space of time methylcholanthrene would have completely disappeared from the body of the animal, and that when the grafts were made there was no likelihood that methylcholanthrene was transferred at the same time as the graft, nor that it was still present in the deep tissues of the treated site.

Nodules which appeared and subsequently regressed are not counted as tumours. This phenonemon does not occur frequently, but it does not seem wise to regard as neoplastic a nodule which lacks the property of persistent growth. In at least one case a nodule on the grafted area has been shown to be an inclusion dermoid.

Tumours which arise below the treated area have been seen. This is evidence of the impossibility of localizing the treated area with exactitude, and would account for an occasional heterotopic tumour.

Difficulties may arise as a result of shrinkage of scar tissue in determining whether a tumour which is in a different place at successive examinations is in fact the same tumour. Similar difficulties may arise from the way in which the mouse is held, owing to the mobility of the skin.

It was regarded as important to remove tumours from the treated area in certain experiments in order to prolong the life of the mouse and give the grafted area the fullest possible chance of developing a tumour.

*Experiment A : Transplantation of Untreated Tail Skin Epidermis to Recipient Area Cut in Carcinogen-treated Skin.*

By the tryptic dissolution of the fine elastic fibres that unite the epidermis to the dermis morphologically intact sheets of " pure " epidermis can be prepared in which the cells are alive, as evidenced by the fact that sheets of epidermis so prepared can be successfully transplanted (Medawar, 1941 ; Billingham and Medawar, 1951).

FIG. 1.—Rate of appearance of tumours (Experiment A) in methylcholanthrene-treated skin after removal of original epithelium and grafting of untreated tail-skin epidermis.

P = papilloma.
C = carcinoma.

Each horizontal line indicates the time of survival of an animal after grafting. The oblique line indicates operative removal of the tumour preceding it.

The object of this series of experiments was to remove the epidermis from the carcinogen-treated area on the right side of the animal's thorax as completely as possible, and then to replace it with sheets of pure epidermis prepared from normal untreated skin elsewhere on the body. Sheets of epidermis prepared from the skin of the tail were selected, since they are thicker and easier to handle than those prepared from the general integument, where the abundant hair follicles interfere with the clean fission of the dermis from the epidermis.

Several thin rectangular Thiersch shavings of even thickness, about 5 mm. × 6 mm., from the skin of the tail were floated on trypsin solution (a Seitz-filtered 0·5 per cent solution of commercial trypsin powder in Ringer-bicarbonate adjusted to pH 7·8) and incubated at 38° C. for 30 minutes. Longer periods were required for thicker shavings. After rinsing in Ringer's solution the skin shavings were carefully spread out, cuticular side down, in a dry sterile Petri dish. The dermis could then be peeled off very easily with fine forceps, leaving the epidermis as an intact sheet.

The epidermis from the carcinogen-treated skin on the animal's thorax was removed simply by cutting a series of very thin Thiersch shavings from it. This operation was greatly facilitated by the rather oedematous thickening due to the carcinogen treatment. A drop of Ringer's solution was placed on the denuded area and on this the pure epidermal grafts were floated. After withdrawal of the excess fluid with a piece of sterile filter-paper the grafts were adjusted in position so as to cover the prepared area as completely as possible. After dusting the operation field with sterile sulphadiazine powder standard dressings were applied.

At the primary and subsequent early inspections satisfactory evidence was obtained that the grafts had taken. By about the 10th day thin cuticular "ghosts" could be peeled away from the grafts to reveal whitish healthy sheets of epithelium, which by their proliferative outgrowth had frequently coalesced.

Since in the preparation of the graft bed the greater portion of the thickness of the dermis was left intact, the bases of the follicles remained behind and so the epithelium of the grafted area was unavoidably of dual origin. Some hairs eventually pierced the surface. It was not possible to distinguish histologically between the transplanted epithelium of tail skin origin, and that originating from truncated follicles left behind in the graft bed.

The results of subsequent periodical inspection of the mice are shown in Fig. 1. One animal did not survive operation up to the time of first tumour appearance, and has not been included. Of the remaining 21 mice, it will be seen that 13 animals developed tumours on the treated area in from 35 to 265 days after grafting, with an average time of 92 $\pm$ 18·5 days. In 2 cases (1270 and 1276) the tumour showed sebaceous differentiation, in one (1234) it was anaplastic. In the remaining 10 animals the tumour was squamous papilloma or carcinoma with keratinization, and appeared on histological evidence to have arisen from the superficial epidermis itself (Fig. 2 and 3). Some of the tumours were found at skin sites where no hair follicles were present.

---

EXPLANATION OF PLATES.

FIG. 2.—Squamous carcinoma (Mouse 1260) arising from the superficial epidermis. × 35.
FIG. 3.—Squamous papilloma (Mouse 1253) arising from superficial epidermis. × 40.
FIG. 6.—Tumour with sebaceous differentiation (Mouse 1279). × 80.
FIG. 7.—Tumour possibly arising from hair follicles (Mouse 1284). × 82.
FIG. 9.—Epidermal papilloma from grafted area (Mouse 1393). Elastic tissue stained. Note altered character of dermis below tumour base. × 45.
FIG. 10.—Epidermal carcinoma from grafted area (Mouse 1346). Elastic tissue stained. Beneath the tumour the dermis has been completely replaced by grafted dermis containing practically no elastic tissue. × 24.
FIG. 11.—Mouse from Experiment G, to show disorientation of hair growing from graft in left dorso-lateral thorax.

2

3

Billingham, Orr and Woodhouse.

6

7

9

Billingham, Orr and Woodhouse.

10

11

*Experiment B : Transplantation of Carcinogen-treated Epidermis to a Recipient Area Cut in Normal Skin.*

The object of this series of experiments was to investigate whether sheets of pure epidermis prepared from the carcinogen-treated skin on the right side of the animal's chest would give rise to tumours when transplanted to a bed cut in normal untreated skin—in our experiments, in the skin on the opposite side of the chest. The sheets of pure epidermis were prepared exactly as in Experiment A

FIG. 4.—Rate of appearance of tumours (Experiment B) in original methylcholanthrene-treated area after removal of thin Thiersch grafts for transplantation elsewhere. Notation and symbols as in Fig. 1.

from thin shavings of skin cut from the treated area. The changes evoked in this skin by the application of the carcinogen greatly facilitated both the cutting of the thin shavings and the subsequent tryptic separation of the dermis from the greatly thickened epidermis.

The resultant pure epidermal grafts were transplanted to a " half-thickness " bed cut in the normal skin on the left side of the animal's chest, by the removal of a series of very thin superficial shavings. As in the previous series of experiments the epithelium resurfacing such a grafted area is unavoidably of dual origin.

It should be added that pure epidermal grafts do unite firmly, and in a functionally adequate way after transplantation to either a freshly cut full-thickness bed, from which all dermal tissue has been removed, or to a similar bed which is already granulating. Unfortunately such an operation field, despite the fact that it has been completely resurfaced by the transplanted epithelium, undergoes progressive contracture, which ultimately results in the approximation of the original margins of the wound. The fate of the transplanted epithelium is at present obscure (Billingham and Medawar, 1951). If, however, sufficient dermal collagen is left behind when the bed is prepared, as was done in our experiments, contracture of the wound does not take place and the pure epidermal grafts manifestly survive.

The results are shown in Fig. 4. Four animals did not survive operation up to the time of first tumour appearance, and have not been included. *No tumours arose on the grafted site in the remaining* 28 *mice.* Tumours appeared on the original treated site in 14 animals. In some cases these tumours were removed to prolong the life of the mouse, and in some of these further tumours occurred, but never on the grafted site. The time range from operation for the appearance of a first tumour was from 37 to 272 days after operation, with an average time of 106 $\pm$ 20 days. Of the 22 tumours in all (including those subsequent to the first in each animal), 3 (1215, 1252, and the third tumour in 1218) showed sebaceous differentiation, in 2 (1207 and the third tumour in 1245) origin from the hair follicles could not be excluded, and one was anaplastic. The remaining 16 tumours appeared histologically to be squamous and horny papillomata and carcinomata of superficial epidermal origin. The survival of the mice after grafting ranged from 41 to 376 days, with an average time of 197 $\pm$ 20·4 days ; one would therefore infer that an adequate opportunity had been given to the grafted epithelium on the left side of the thorax to reveal its neoplastic potency if present.

*Experiment C : Transplantation of Thin Thiersch Grafts of Carcinogen-treated Skin to a Recipient Area Cut in Normal Skin.*

In these experiments 4 thin Thiersch grafts were cut from the treated skin on the right side of the animal's chest and transplanted to a full thickness bed cut down to the vascular fascial plane in the untreated skin of the opposite side of the chest, the aggregate area of skin so transplanted being 1 to 2 sq. cm., depending on the size of the animal. Whereas in most animals the grafts were transplanted to a common bed, in a few each of the 4 grafts was transplanted to a separate full-thickness bed, the individual beds being separated by small stretches of intact skin. As some of these grafts eventually bore hairs, it seems possible that the operative aim was not fully achieved, and that either the bed or the graft must have contained hair bulbs.

The results are shown in Fig. 5. Three animals (two single-bed and one multiple-bed graft) did not survive operation up to the time of first tumour appearance, and have not been included. In the remaining 15 mice no tumours appeared on the grafted sites, although in one instance (1284) the tumour arose close to the boundary between the two sites and was accurately localised only after histological examination. There was one other lesion on a grafted site (1223) which proved on histological examination to be an implantation epidermoid cyst. Tumours appeared on the original treated area in 7 animals, and some were removed in order to prolong the survival time of the mouse. The mean time after operation of tumour appearance was 38 ± 7·5 days. Of the 9 tumours in all, 2 (1223 and the second tumour of 1279) showed some sebaceous differentiation (Fig. 6) and in one (1284) an origin from hair follicles could not be excluded histologically (Fig. 7); the remaining 6 showed origin from the superficial epidermis, and 2 of these were arising in a part of the skin from which hair follicles were absent.

Fig. 5.—Rate of appearance of tumours (Experiment C) in original methylcholanthrene-treated area after removal of thin Thiersch grafts.

× (in Mouse 1298) = mammary carcinoma.

ό (in Mouse 1223) = implantation epidermoid cyst in grafted area.

Remaining notation as in Fig. 1.

*Experiment D: The Transplantation of Thick Thiersch Grafts of Carçinogen-treated Skin to a Recipient Area Cut in Normal Skin.*

Apart from the fact that the grafts were thicker than those used in the experiments described in (C) above no further comment is necessary. In every case the grafts were transplanted to a common full-thickness bed.

The results are shown in Fig. 8. One animal did not survive operation up to the time of first tumour appearance, and has not been included. In the remaining 30 mice tumours appeared on the grafted site in 5 animals, and on the original treated site in 5 animals. Two animals bore a tumour at each site. The time after operation of appearance of tumours at the grafted site ranged from 21 to 90 days, with a mean time of 53 ± 11·5 days. The corresponding values for tumours on carcinogen-treated sites are 21 to 392 days (mean 186 ± 75). In

3 of the tumours on grafted sites there was histological evidence that the entire thickness of the recipient dermis had been removed in the region where the tumour arose, and replaced by more or less the entire thickness of the transplanted dermis (Fig. 9 and 10). This is regarded as a point of some importance in finding

FIG. 8.—Rates of appearance of tumours (Experiment D) in methylcholanthrene-treated donor area and untreated recipient area after transference of thick Thiersch grafts.

Letters above the life-line = tumours on original treated area.
Letters below line = tumours on grafted area.

Notation otherwise as in Fig. 1.

an explanation for the discrepancy between this experiment and Experiment C, and the fact that the grafts were cut thicker may also account for the lower yield of tumours on the original treated site. None of the tumours showed evidence of origin from hair follicles or sebaceous glands.

*Experiment E : The Exchange of Full-thickness Grafts between the Treated and Untreated Sides of the Chest.*

Standard " pinch " grafts were cut from the normal skin and the treated skin on the left and right sides respectively of the animal's chest. After trimming off all remnants of the muscle and fatty layers each graft was transplanted to the bed on the opposite side of the body, so that the graft of carcinogen-treated skin was transplanted to a defect in normal skin and *vice versa*. Under these conditions the grafts were unavoidably rather " open " fits, an annulus of exposed raw tissue lying between the rim of the graft and the margin of its bed. The healing of these grafts was perfectly satisfactory, re-surfacing of the annulus of raw tissue being brought about by the outward migration of epithelium from the graft and the inward migration of epithelium from the margin of its bed. A slight modification of the standard dressings was necessary in these bilaterally operated animals. Instead of applying separate rectangles of tulle gras over the two operation fields, a 3-inch strip of rather coarser mesh tulle gras (prepared by impregnating $\frac{3}{8}$-inch open-wove bandage with vaseline) was wound round the entire thorax so that it covered both areas, after which a length of plaster was applied.

Of the 14 mice which survived operation up to the time of first tumour appearance, 3 developed tumours (in 55, 55 and 85 days after operation), all on the original treated site. In 2 of these removal of the tumour was followed by the appearance of a further tumour. No tumours appeared on the site which had received carcinogen-treated grafts. Six of the mice were still alive 211 days after operation. Only one of the tumours showed histological evidence of origin from hair follicles and sebaceous glands ; 4 of the 5 were malignant. Histological examination of both sites in a mouse which died 12 days after operation showed that while the graft of treated skin to the untreated site was apparently fully viable and had " taken", the reciprocal graft of untreated skin to the treated site was necrotic throughout its thickness.

*Experiment F : Reflection of a Full-thickness Flap of Skin from the Treated Area, followed by Resuturing into its Original Position.*

In the experiments so far described, as in those of the following sections, " free " skin grafts have been used, i.e., grafts which have been completely freed from all connections with the body. Such grafts have perforce to suffer a period of ischaemia before the re-establishment of a vascular supply after transplantation. The object of this series of experiments was to effect the partial but not complete interruption of the vascular system of the graft. This was effected by making incisions through the skin down to the body wall along three sides of a rectangle, about 1·5 cm. cephalo-caudally × 2 cm. dorso-ventrally, which included practically the entire treated area. The fourth side of the rectangle, which ran along the dorsal midline, was not incised, being left as a pedicle on which the graft (in this case including the panniculus carnosus) was reflected from the body wall. After this it was replaced and sutured in its original position with interrupted fine silk sutures. Standard dressings were then applied. The healing of these flap grafts was uneventful.

Of 6 mice which survived operation up to the time of first tumour appearance, 2 developed tumours (in 53 and 57 days after operation). At the time of writing 2 mice are alive 161 to 168 days after operation, without tumours. Both tumours appeared to arise from the superficial epidermis ; one was a papilloma, the other a carcinoma.

*Experiment G : Transplantation of a Full-thickness Graft of Treated Skin to a Recipient Area Cut in Normal Skin.*

This series is in effect a repetition of series (E), except that no attempt was made to transplant a graft of normal skin to the treated area. Every attempt was made to cut as large a graft as possible from the treated area, and to ensure that it fitted exactly into the bed cut to receive it in the normal skin on the left side of the animal's chest. These grafts were readily detected for many months by the disorientation of hairs (Fig. 11).

Of 23 mice which survived operation up to the time of first tumour appearance, 3 developed tumours (in 44, 102 and 162 days) on the original treated area, and 3 (in 46, 84 and 98 days) on the grafted area. One animal, included in both these groups, bore tumours on both sites. At the time of writing 17 mice are still alive, having survived operation for periods of 126 to 168 days.

FIG. 12.—Rate of appearance of tumours (Experiment H) in methylcholanthrene-treated skin after detachment and re-implantation. Notation as in Fig. 1.

*Experiment H : The Re-implantation of a Pinch Graft Cut from the Carcinogen-treated Skin Back into its Own Donor Site.*

In these experiments a large pinch graft was cut from the carcinogen-treated skin, all remnants of the muscle and fatty layers trimmed off, and the graft re-implanted into the defect from which it had been taken. Owing to the natural gape of the wound after cutting and the slight contraction of the graft it was a rather " open " fit. Nevertheless these grafts healed-in satisfactorily.

The effect of this procedure was greatly to augment the carcinogenic process in the group of mice tested. The results are shown in Fig. 12. Of 14 mice which survived operation up to the time of first tumour appearance, 12 developed tumours in 39 to 90 days (mean 57 $\pm$ 5 days). At 90 days the experiment was terminated by killing the remaining mice. In addition to their high incidence and early onset the tumours in individual animals were strikingly larger than in the other experiments, and in 5 instances rapidly spread or coalesced to involve practically the whole of the treated and grafted area. Histologically 2 of the tumours (3956 and 3983) might have originated in hair follicles, and one (3963) showed sebaceous differentiation; the remaining 9 were apparently derived from the superficial epidermis. Seven were papillomata, and 5 carcinomata. In a further group of animals in which the treated area was grafted with normal skin from the left thorax, tail, or ear, the grafts took satisfactorily, but no similar acceleration of tumour formation was observed. This latter group has not yet been under observation long enough, however, to give the final results.

### DISCUSSION.

The present results offer some support to the view that the effective carcinogenic action of methylcholanthrene is not limited to the epithelium itself, but that the changes in the deeper tissues are of great importance. The most striking results obtained seem to be that no tumours arose on the grafted areas of Experiments B and C. If this finding is to be taken at its face value it implies that the treated epidermis no longer retained potential neoplastic properties after its transference to the untreated site. It is, therefore, crucial to attempt to decide whether these transplants were in point of fact effective. Clinical examination suggested that the grafts had taken adequately. To confirm this impression, small sheets of carcinogen-treated epidermis were transplanted to the centre of a large full-thickness bed. Under these conditions it was possible to observe directly the outgrowth of epithelium from the margins of the graft over the granulating surface. This is regarded as unequivocal proof of the viability of transplanted carcinogen-treated epithelium. It is not unreasonable, therefore, to believe that it survived in the conditions of Experiments B and C.

If the results obtained in Experiment A are subjected to the simplest explanation, the conclusion would be that it is not necessary for the epithelium itself to be directly acted upon by methylcholanthrene at all. We are fully alive to the fact that such a direct interpretation of the latter result is not fully justified at present, inasmuch as the deeper parts of the hair follicles were left *in situ* when the graft beds were prepared. It is now accepted that regeneration of epidermis can occur by outgrowth from the lining of hair follicles, and that new hair follicles and sebaceous glands can be differentiated from the superficial epidermis. The presence of hair follicle or sebaceous gland structure in tumours cannot therefore be used to provide conclusive proof that a tumour has originated from these sources, and similarly their absence cannot be invoked decisively to prove that tumours have originated from other sources. With appropriate reservations, however, we feel entitled to point out that the majority of the tumours in Experiment A showed no evidence of structure other than that of simple epidermis, and we are satisfied that the grafts of tail skin epidermis did in fact " take", and that there is a strong probability that it was this epithelium in which the neoplastic change took place in most cases.

In experiments where the graft included dermis as well as epidermis, the location of the tumours on grafted sites appeared to depend on the amount of dermis which had been transferred. Thus in the thick Thiersch grafts of Experiment D some tumours were obtained on the grafts, while none occurred on the thin Thiersch grafts of Experiment C. In Experiment D the tumours on the grafted area tended to appear relatively early in the experiment, and it is possible that they may have been grafted *as tumours* (not yet visible to the naked eye). In Experiments E and G (full thickness grafts) there is little difference in the incidence of tumours as between treated and untreated sites, but the relatively low total yield of tumours suggests that in some way the carcinogenic stimulus must have been less potent in these experiments than in some of the others.

The results of Experiment H would lead one to believe that the carcinogenic potentialities inherent in methylcholanthrene-treated dermis are enhanced when it is divided and replaced *in situ* so that a healing reaction takes place. We have never seen experimental skin tumours develop so rapidly and extensively as they did in this group of mice ; in some of the animals tumours were already beginning to appear before the post-operative inspection of grafts was completed. In the light of this experiment a new explanation can be offered of the well-known results of Deelman (1927), who found that tumours appeared unusually rapidly in the track of an incision made in tarred skin, and of the similar findings of Friedewald and Rous (1950) in methylcholanthrene-treated rabbit's ears in which healing of punch holes had taken place.

None of our other experiments have given the type of result obtained in Experiment H. An important point may be that this was the only experiment in which both the implanted and recipient connective tissues had been subjected to the action of methylcholanthrene, and the former completely severed from its blood supply (Experiment F). It may be that the healing reaction between two carcinogen-damaged connective-tissue surfaces accelerated the development of decisive carcinogenic conditions.

In those experiments (B, C, D and G) in which the donor site was left to re-surface itself from natural sources, i.e., without a skin graft, the new epidermis would be derived from ingrowth of the surrounding epidermis or by migration from the transected hair follicles, or from both sources. In Experiment B in particular care was taken to try to remove all the treated epidermis, so that only the hair follicles would be available as a source of epithelium which had been exposed to the action of the carcinogen. It may therefore be argued with some justification that epithelium of such derivation was the source of the tumours. This, however, would imply that epithelium of the hair follicles had been altered into a state of latent neoplasia by the action of the carcinogen, but had at the same time retained the power of re-differentiating into superficial epidermis. In our opinion this view is unnecessarily complicated, and on the principle of minimal hypothesis, attention should first be given to the possibility that epidermal carcinoma may be determined by growth and multiplication of epidermal cells, which are dependent for their nutritional requirements on a dermis and subcutis which have been altered in structure and function by a suitable agent. That there are early histologically demonstrable changes in epidermis treated with carcinogens is not denied, but it has still to be shown that they are relevant to actual tumour formation, especially in the light of the results of Experiment B.

If the essential primary carcinogenic change is in the sub-epidermal tissues, it would seem reasonable to expect that the tumour incidence would be secondarily influenced by the rate of epithelial proliferation. Considerations of this sort offer a tenable explanation of the phenomenon of so-called co-carcinogenesis (Berenblum, 1941, 1944). Croton oil, the best known co-carcinogen, produces rapid and active hyperplasia of the epidermis, but this only exceptionally goes on to tumour production unless the skin has previously received treatment with a carcinogenic agent. The interval between the application of carcinogen and co-carcinogen can be a very long one, and in the meantime the epidermis reverts to a histological structure which is indistinguishable from normal. Clearly there must be some small proliferative activity during this period to replace the effects of attrition and loss of cells. It is suggested that the integrity of the dermis is sufficient to sustain this, but that when the rate of proliferation of epidermis is stepped up by croton oil, the deficiencies of the dermis are brought to light, and tumours begin to appear.

To the best of our knowledge grafting experiments of this type have not previously been reported in relation to experimental carcinogenesis. Silberberg, Silberberg and Hulbert (1948) have studied the effect of 20-methylcholanthrene on the transplantability of mouse skin, and have reported that treatment of skin with this agent previous to transplantation intensified the growth processes in the grafts, but unless the transplanted skin had acquired neoplastic properties their transplantability was decreased as compared with normal skin. Though the technique used involved orthotopic transplantation, their object was to study the early healing processes in the grafts which were removed at a very early stage for histological examination before they had entered the phase of tumour production. Their experiments and ours are, therefore, of limited relevance to each other.

It is premature to draw general conclusions from the work now reported, but if the failure to obtain tumours in transplanted carcinogen-treated epidermis is confirmed by future study, it must be taken into account in attempts to explain the mechanism of carcinogenesis. The crucial issues appear to be whether such grafts take, and whether the grafted epithelium survives long enough to give neoplastic change an opportunity to manifest itself. We believe that the first of these questions can be answered in the affirmative ; the second question raises implications which cannot yet be fruitfully discussed, but which would seem to be applicable also to epithelium left undisturbed in its original site of carcinogenic treatment.

### SUMMARY.

A small area of skin was treated in mice with 20-methylcholanthrene in weekly applications for 12 weeks, this being a fully effective carcinogenic stimulation. Such mice, provided they had not already developed visible tumours, were used for transplantation experiments.

Carcinogen-treated epidermis, and thin Thiersch grafts of carcinogen-treated skin, did not yield tumours when transplanted orthotopically to untreated body sites.

Thick Thiersch grafts and whole thickness grafts of carcinogen-treated skin yielded a small number of tumours after transplantation.

Tumours were obtained in considerable number when a denuded carcinogen-treated area was re-surfaced with untreated epidermis.

Re-implantation of whole-thickness grafts of carcinogen-treated skin in the beds from which they were cut resulted in an enhanced rate and extent of carcinogenesis.

This work was supported by the British Empire Cancer Campaign and its Birmingham Branch. We have also to acknowledge the help of Miss Jean Morpeth.

### REFERENCES.

BERENBLUM, I.—(1941) *Cancer Res.*, **1**, 807.—(1944) *Arch. Path.*, **38**, 233.
BILLINGHAM, R. E., AND MEDAWAR, P. B.—(1951) *J. exp. Biol.*, **28**, 385.
*Idem*, ORR, J. W., AND WOODHOUSE, D. L.—(1950) *Nature*, **166**, 1080.
DEELMAN, H. T.—(1927) *Brit. med. J.*, i, 872.
FRIEDEWALD, W. F., AND ROUS, P.—(1950) *J. exp. Med.*, **91**, 459.
GLUCKSMANN, A.—(1945) *Cancer Res.*, **10**, 255.
HANDLEY, W. S.—(1931) 'The Genesis of Cancer.' London (H. K. Lewis).
HOWES, E. L.—(1946) *Cancer Res.*, **6**, 298.
KREYBERG, L.—(1929) *Arch. path. Anat.*, **273**, 367.
MEDAWAR, P. B.—(1941) *Nature*, **148**, 783.
ORR, J. W.—(1937) *J. Path. Bact.*, **44**, 19.—(1938) *Ibid.*, **46**, 495.
PULLINGER, B. D.—(1940) *Ibid.*, **50**, 463.
SALAMAN, M. H., AND GWYNN, R. H.—(1951) *Brit. J. Cancer*, **5**, 252.
SILBERBERG, M., SILBERBERG, R., AND HULBERT, B.—(1948) *Arch. Path.*, **45**, 722.